# Improved preservation of hearts from old donors during static cold storage by the senomorphic agent ruxolitinib

**DOI:** 10.3389/frtra.2026.1917058

**Published:** 2026-10-28

**Authors:** Lars Saemann, Belma Kozar, Sabine Pohl, Anna Hoffmeister, Lena Wernstedt, Yao Gong, Andreas Simm, Gábor Szabó

**Affiliations:** 1 Department of Cardiac Surgery, University Hospital Halle, University of Halle, Halle (Saale), Germany; 2 Department of Cardiac Surgery, University Hospital Heidelberg, Heidelberg, Germany

**Keywords:** aging, frailty, gender medicine, heart transplantation, intrinsic capacity, ruxolitinib, senescence-associated secretory phenotype, senotherapy

## Abstract

**Purpose:**

Hearts from old donors are more likely to develop primary graft dysfunction, presumably due to reduced tolerance to ischemia/reperfusion injury, partially driven by proinflammatory cytokines. Accumulating senescent cells secrete the senescence-associated secretory phenotype (SASP), including proinflammatory cytokines, potentially intensifying IRI. Senomorphic agents block the SASP. We investigated the effect of the senomorphic agent ruxolitinib on the contractility of hearts from old donors.

**Methods:**

We procured hearts from 24-month-old male and female C57BL6/J mice and preserved them by static cold storage (SCS) for 2 h using histidine-tryptophan-ketoglutarate (HTK) solution, without or with ruxolitinib (HTK + Ruxo), and measured contractility before and after SCS. In the Control group, hearts were not preserved by SCS (*N* = 5 per Group and sex). We determined SASP-related cytokine release after SCS, JAK pathway, and nitrotyrosine levels.

**Results:**

Especially in hearts from female mice (developed pressure, DP: 111 ± 17 vs. 44 ± 10%, *p* < 0.05) and (minimal slope of pressure decrement, dp/dt_min_: 103 ± 13 vs. 78 ± 8%), but also in hearts from male mice, the ventricular function (DP, 71 ± 9% vs. 47 ± 18%; dp/dt_min_, 87 ± 7% vs. 54 ± 10%) was higher in the HTK + Ruxo vs. the HTK group. IL-1*α*, IL-7, and nitrotyrosine levels were lower in the HTK-Ruxo group, independent of sex, and TNF*α* was lower only in the hearts of female mice. The phosphorylation of JAK2 was increased in the hearts of male mice in the HTK group.

**Conclusion:**

The senomorphic agent ruxolitinib improves contractility and modifies the SASP-related cytokine release during SCS of hearts from old donors in mice and is more effective in female hearts.

## Introduction

Based on the organ shortage, hearts from donors with extended criteria, such as old age, are increasingly accepted for heart transplantation (HTX) in Europe (1). Unfortunately, old age is a risk factor for primary graft dysfunction (PGD) after HTX, which is associated with a poor prognosis for the patient (2). Female donor sex can also be a risk factor for PGD, according to a meta-analysis by Buchan et al. (3). Some achievements have been made to preserve hearts donated from old donors by *ex vivo* blood perfusion (4). However, the use of hearts from older donors, maintained by *ex vivo* blood perfusion in clinical practice, is not routine, and static cold storage (SCS) remains the most convenient and cost-effective method for organ preservation. Effective myocardial protection during SCS is crucial for preventing ischemia/reperfusion injury (IRI), which is one of the underlying causes of PGD and may be even more important in hearts from elderly donors (5).

By age, the number of senescent cells increases in the tissue. Senescent cells secrete a profile of biomolecules called the senescence-associated secretory phenotype (SASP), which includes proinflammatory cytokines. Such cytokines are known to partially mediate or intensify IRI (6). The senomorphic agent ruxolitinib, a Janus kinase (JAK) 1 and 2 inhibitor, blocks SASP secretion and may therefore be a protective agent for organ preservation (7, 8). We have already shown in vascular IRI that ruxolitinib reduces IRI-mediated endothelial dysfunction and is more effective in female subjects (9). However, nothing is known about ruxolitinib in organ preservation. Taking all factors into account, we investigated the effect of ruxolitinib as a supplement to SCS in hearts from elderly donors.

## Material and methods

### Animals and anesthesia

The studies involving animal participants were reviewed and approved by the Institutional Ethical Committee for Animal Experimentation of the Medical Faculty of the Martin-Luther-University Halle Wittenberg (approval number: k2bM7). The animals received humane care. We used male and female C57BL/6J mice (Janvier, France) aged 24 months for the experiments. We injected 200 µL (5,7 iU) of heparin intraperitoneally and anesthetized the mice rapidly with 5% isoflurane in oxygen. After having checked unconsciousness by the pedal reflex test, the mice were euthanized by cervical dislocation.

### Study groups and animal experimental procedure

Immediately after euthanization, the thoracic cavity was rapidly opened by bilateral thoracotomy, and the heart was harvested and submerged in ice-cold carbogenized Krebs-Henseleit-Solution (KHS). Under microscopic vision, the aorta was cannulated, connected to a perfusion machine, and perfused in Langendorff fashion through the ascending aorta. After a stabilization period of 10 min, a left-ventricular balloon catheter was inserted through the mitral valve to perform a baseline contractile evaluation (Figure 1). Then the hearts were flushed with 2 mL of cold Histidine-Tryptophane-Ketoglutarate (HTK, Köhler Chemie, Bensheim, Germany) preservation solution (HTK-group; female: *n* = 5; male: *n* = 5) or HTK with added ruxolitinib (HTK + Ruxo-group; female: *n* = 5; male: *n* = 5) and preserved by SCS at 4 °C for 2 h. The hearts were then reconnected to the perfusion machine to assess contractile function after SCS. In a Control-group (female: *n* = 5; male: *n* = 5), the hearts did not undergo cardioplegic arrest and SCS, but the second functional evaluation was performed immediately after the first. In a technical control group (HTK flush; female: *n* = 5; male: *n* = 5), the hearts were flushed with HTK solution but not stored for 2 h to determine the sole effect of flushing.

**Figure 1:**
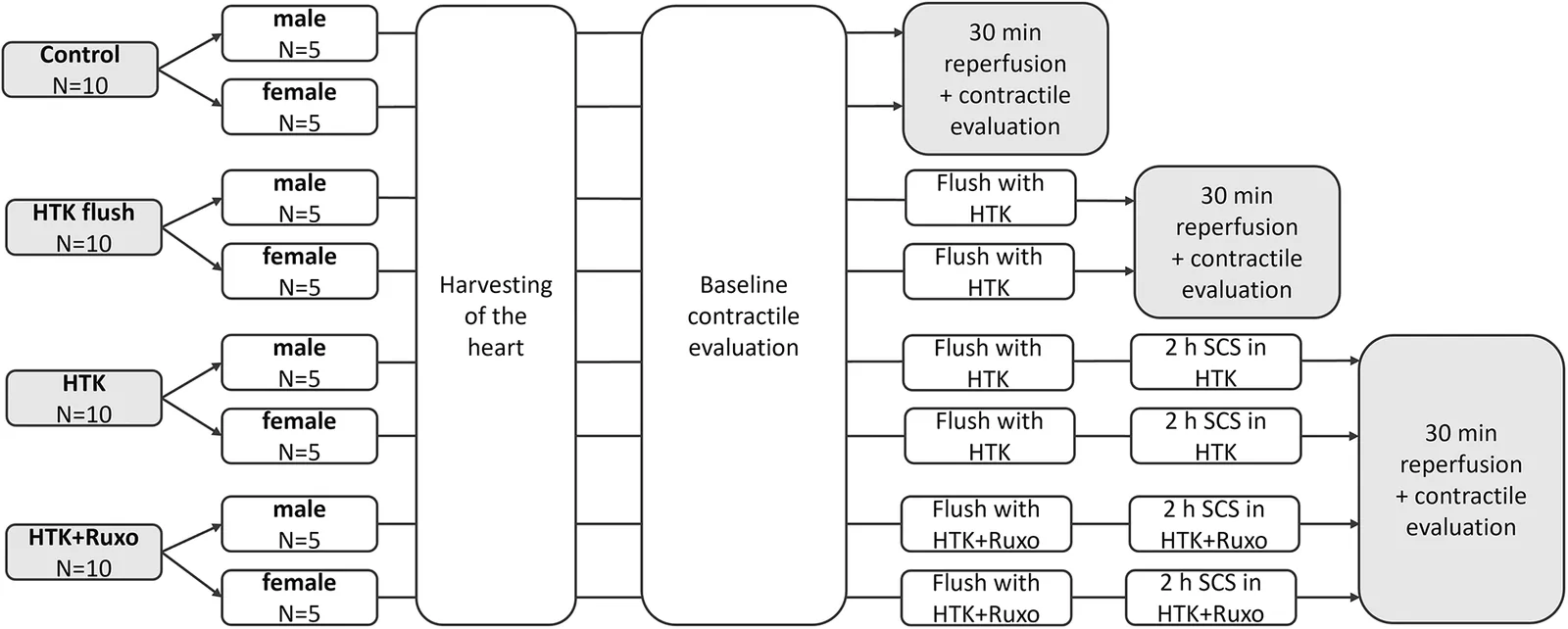
Groups. HTK: Histidine-Tryptophane-Ketoglutarate. Ruxo: Ruxolitinib. SCS: static cold storage.

In the HTK + Ruxo-group, ruxolitinib was dissolved in 20 µL DMSO and added to 2 mL of HTK preservation solution to reach a final concentration of 10nM. As a vehicle, 20 µL DMSO without ruxolitinib was added to the preservation solution in the HTK group. The experimental surgeon was blinded to whether vehicle or ruxolitinib was added to the preveration solution.

### Reperfusion and functional analysis

The perfusion machine consisted of a heat exchanger, a micro-membrane oxygenator, a bubble trap, a roller pump, and tubing. The membrane oxygenator was connected to a Carbogen supply to enable continuous gas exchange in the perfusate, which was recirculated through the heart. Using the ventricular balloon catheter, we determined the contractile parameters, developed pressure (DP), maximal pressure increment (dp/dt_max_), and the contractility index (CI), as well as the diastolic parameter, maximal pressure decrement (dp/dt_min_). The CI is calculated by dividing dp/dt_max_ by the maximum pressure at the time point of dp/dt_max_. It is consequently a parameter that reflects contractility corrected for a possible interaction between dp/dt_max_ and maximal pressure.

### SASP-related cytokines

We determined the following SASP-related cytokines from the very first coronary effluent using a Luminex-based method after the onset of reperfusion: IL-1*α*, IL-1β, IL-6, IL-7, IL-10, IL-13, IL-15, TNF-α, CCL2, CCL3, CCL5, CCL8, CCL11, CD27, CXCL5, CXCL9, ICAM1, CRP, G-CSF, GM-CSF, GRO-α, IP-10, M-CSF, and VEGFA. Procedural details are shown in the supplemental. Values below the limit of detection (LOD) were included as LOD/2, as recommended by the manufacturer.

### Tissue collection, western blot, and immunohistochemistry

Samples of left-ventricular myocardial tissue were frozen in liquid nitrogen and stored at −80 °C. We determined the levels of JAK2, pJAK2, STAT3, pSTAT3, Akt, pAkt, mTOR, and *p*-mTOR by western blotting. In addition, we conserved tissue samples in paraformaldehyde and embedded them in paraffin. Then, immunohistochemical staining for nitrotyrosine (HM5001, HyCult Biotech, Uden, The Netherlands, 1:200 dilution, Citrate pH 6.0), a marker of nitrosative stress, and endothelial nitric oxide synthase (eNOS, AB30007, Abcam, Cambridge, UK, 1:100 dilution, Tris-EDTA pH 9.0) was performed. Procedural details on western blotting and staining are shown in the supplement.

### Statistics

Results are expressed as mean ± standard deviation. We tested for homogeneity of variances using the Levene test in IBM SPSS Statistics for Windows (Version 25.0, IBM Corp., Armonk, NY, USA). The data were analyzed using a one-way analysis of variance (ANOVA) with Fisher's LSD adjustment of *p*-values for homogeneity of variances and the Games-Howell adjustment for inhomogeneity of variances. A value of *p* < 0.05 was considered statistically significant. A two-way ANOVA was used to analyze the between-subject effects of treament (HTK or HTK + Ruxo)*sex. In the cytokine comparison, *p*-values were Benjamini-Hochberg-corrected.

## Results

### Ventricular function

Ventricular function did not change compared with the baseline measurement in the Control group. The contractility, as well as ventricular relaxation, was comparable between the HTK-flush and the Control group in hearts from both sexes. In hearts from female mice in the HTK group, the developed pressure (dp/dtmax) and CI were significantly lower than in the Control or HTK-flush group (Figure 2A). The DP and dp/dt_max_ in the HTK + Ruxo group were significantly higher than those in the HTK group. The dp/dt_min_ was also higher in the HTK + Ruxo group than in the HTK group, but not significantly. In hearts procured from male mice, the relative DP, dt/dt_max_, CI, and dp/dt_min_ were higher in the HTK + Ruxo group compared to the HTK group (Figure 2B). However, the improvement was significant only for dp/dt_min_. The F-value for treatment, but not for sex, was relatively high for all functional parameters (Table 1). The HTK + Ruxo treatment also showed a significant effect across all functional parameters. However, neither sex nor the interaction of treatment*sex did. Thus, sex also did not significantly affect the treatment effect.

**Figure 2:**
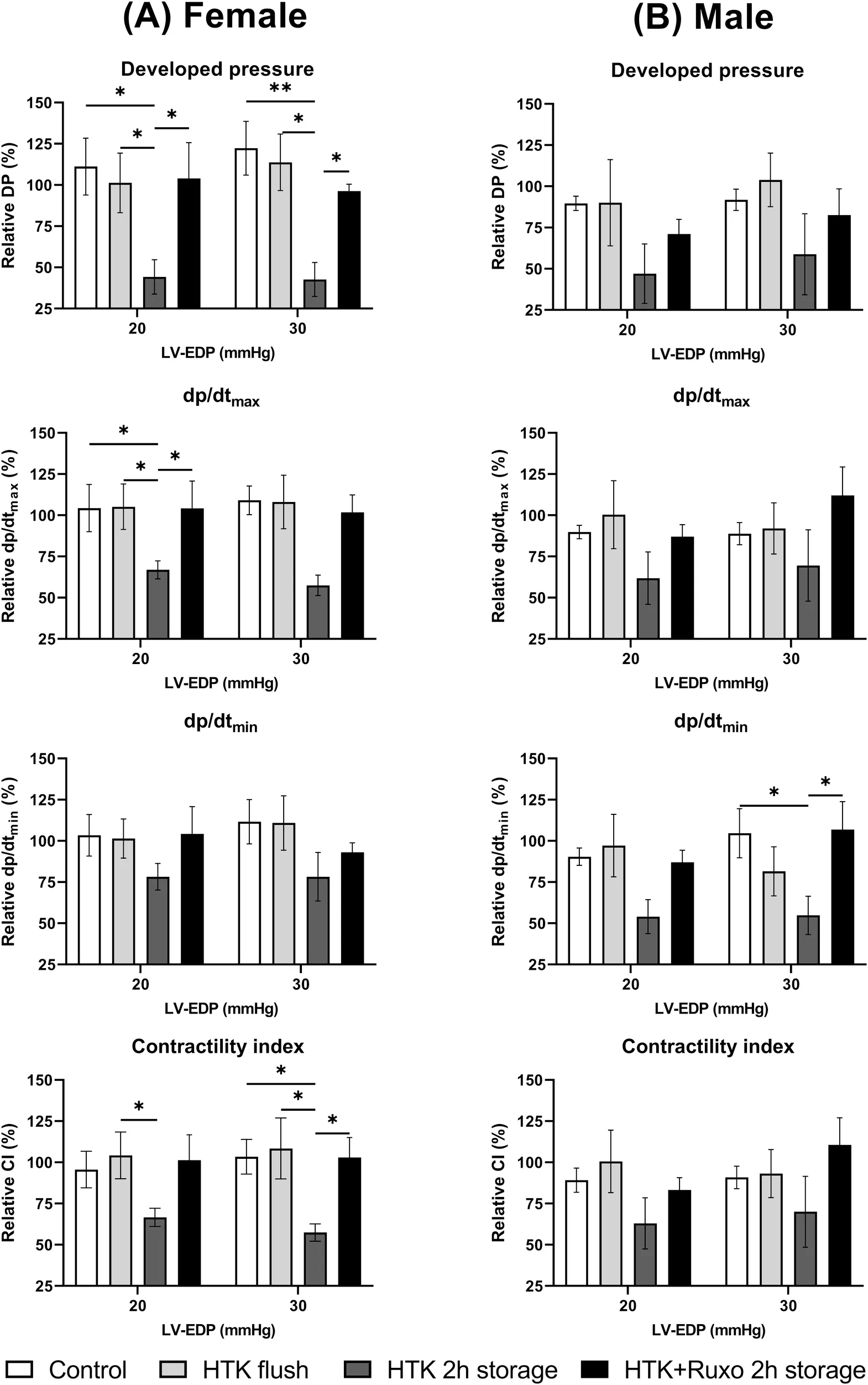
Left-ventricular contractility. **(A)** Hearts from female mice. **(B)** Hearts from male mice. CI: Contractility index. DP: Developed pressure. dp/dt_max_: Maximal pressure increment. dp/dt_min_: Maximal pressure decrement. EDP: End-diastolic pressure. HTK: Histidine-Tryptophane-Ketoglutarate. LV: Left ventricle. Ruxo: Ruxolitinib. *N* = 5/group and sex. *p < 0.05. **p < 0.001.

**Table 1:** Between-subjects effects.

DP @ EDP = 20 mmHg			
Source	F	Sig.	Partial Eta2
Treatment	7,142	,017	,309
Sex	,918	,352	,054
Treatment * Sex	1,291	,273	,075
DP @ EDP = 30 mmHg			
Treatment	6,105	,025	,276
Sex	,006	,940	,000
Treatment * Sex	,909	,355	,054
dp/dt_max_ @ EDP = 20 mmHg			
Treatment	6,387	,022	,285
Sex	,806	,383	,048
Treatment * Sex	,242	,630	,015
dp/dt_max_ @ EDP = 30 mmHg			
Treatment	8,210	,011	,339
Sex	,538	,474	,033
Treatment * Sex	,004	,952	,000
dp/dt_min_ @ EDP = 20 mmHg			
Treatment	6,985	,018	,304
Sex	3,427	,083	,176
Treatment * Sex	,098	,758	,006
dp/dt_min_ @ EDP = 30 mmHg			
Treatment	6,621	,020	,293
Sex	,137	,716	,008
Treatment * Sex	2,047	,172	,113
CI EDP = 20 mmHg			
Treatment	5,341	,034	,250
Sex	,831	,375	,049
Treatment * Sex	,371	,551	,023
CI EDP = 30 mmHg			
Treatment	8,203	,011	,339
Sex	,449	,512	,027
Treatment * Sex	,028	,869	,002

*indicates the between-subject analysis of treatment and sex.

### SASP-related cytokine release

In hearts from female mice, IL-10, IL-1*α*, IL-7, IL-13, TNF-α, CCL5, and CD27 were released less in the HTK-Ruxo group as compared to the HTK group (Figure 3). In hearts from male mice, although the differences were small, CXCL5 was released less, and CD27 was released more in the HTK-Ruxo group. The release of CCL11 was also reduced in the hearts of male mice in the HTK-Ruxo group. The release of CXCL5, ICAM1, CCL2, and CCL8 was comparable between both groups in the hearts of female mice, and of CRP, TNF-α, IL-7, G-CSF, and CCL2 was comparable in the hearts of male mice. The release of CRP and CCL11 was increased in the hearts of female mice and of CCL8 in the hearts of male mice. The concentrations of IL-6, IL-13, and G-CSF were barely above the detection limit and were unlikely to change with ruxolitinib treatment. The concentrations of GM-CSF, GRO-α, IL-1β, IL-15, IP-10, M-CSF, CXCL9, CCL3, and VEGFA were analyzed but were below the detection limit and therefore very low or not apparent.

**Figure 3:**
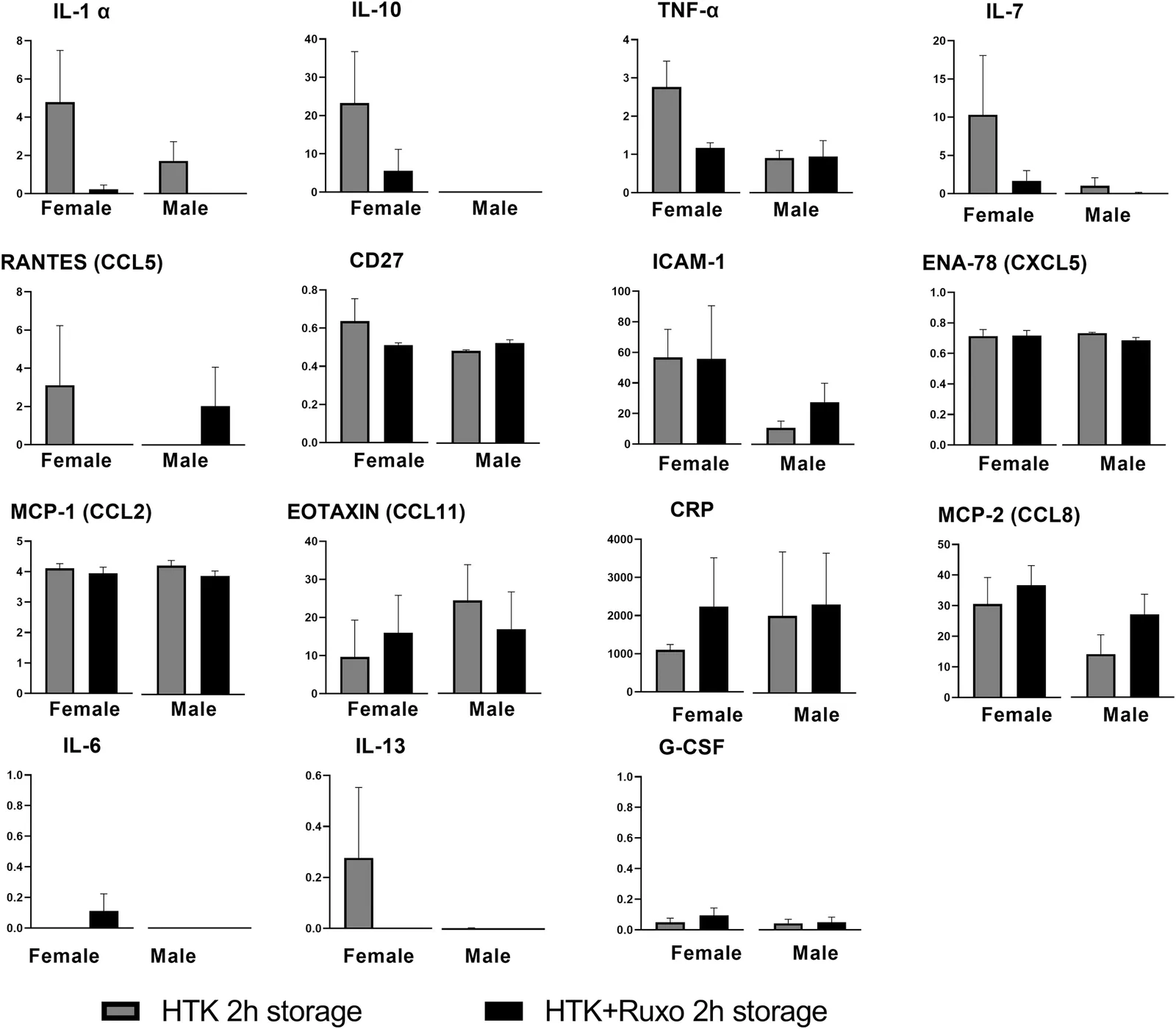
SASP-related cytokine release. HTK: Histidine-Tryptophane-Ketoglutarate. Ruxo: Ruxolitinib. *N* = 3-5/group and sex.     *P*-values were Benjamini-Hochberg-corrected.

### Pathways

#### JAK2/STAT3 pathway

Western blot analysis revealed that phosphorylation of JAK2 was non-significantly increased in the HTK group in hearts from male mice (Figure 4). In the HTK + Ruxo group, JAK2 phosphorylation was much lower and comparable to that in Control hearts. In the hearts of female mice, JAK2 phosphorylation did not differ significantly across groups. The phosphorylation of STAT3 was higher in all groups compared to Control in hearts from both sexes.

**Figure 4:**
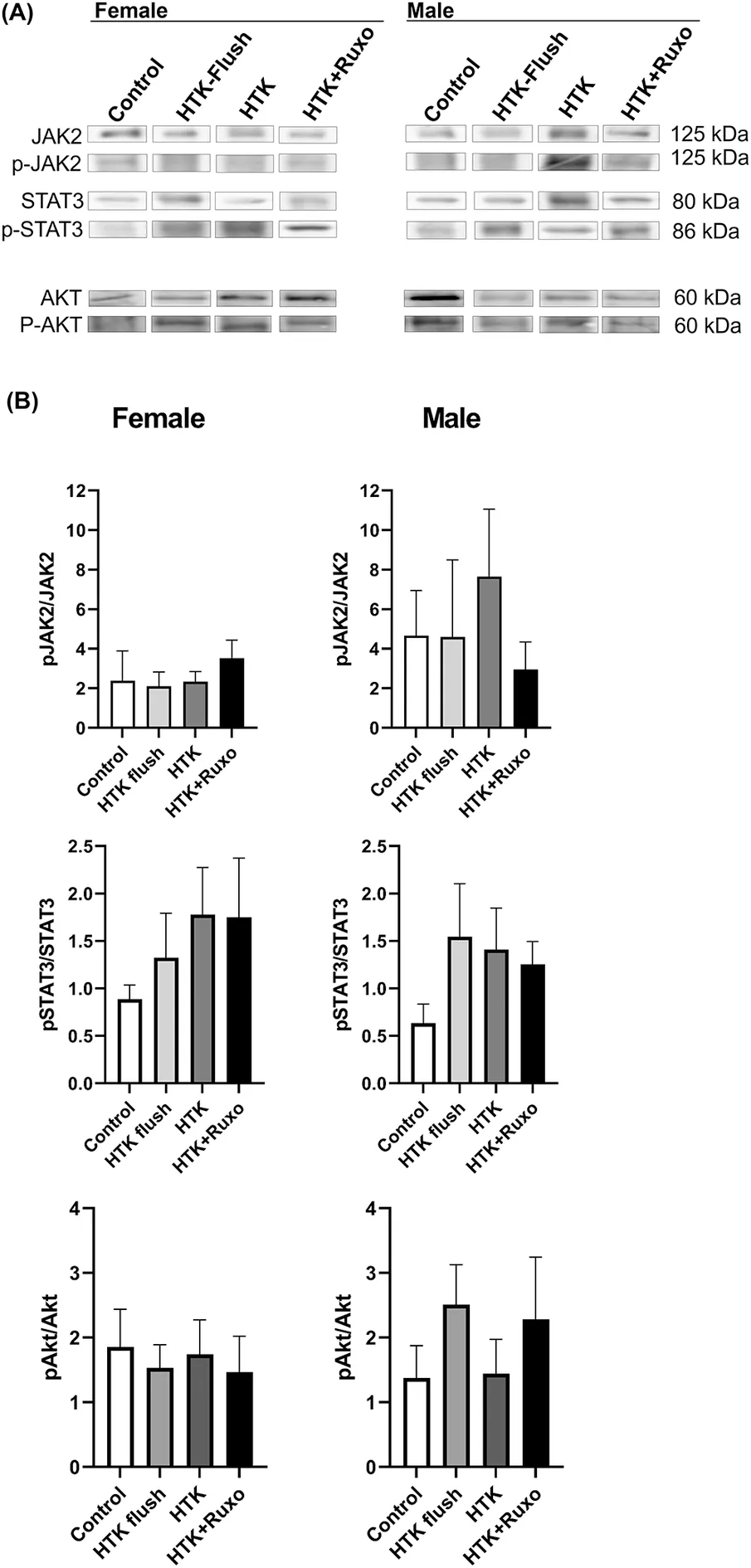
Western blot analysis. **(A)** Representative bands. **(B)** Quantitative analysis. HTK: Histidine-Tryptophane-Ketoglutarate. JAK2: Januskinase 2. STAT3: signal transducer and activator of transcription 3. mTOR: Mammalian target of rapamycin. Ruxo: Ruxolitinib. *N* = 5/group and sex.

#### Akt pathway

Akt phosphorylation was comparable across all groups in hearts from female donors. In hearts   from male donors, pAkt levels were elevated in the HTK + Ruxo group and, surprisingly, in the HTK flush group as well.

### Nitrosative stress and eNOS

Nitrotyrosine was expressed higher in the HTK group in hearts from donors of both sexes (Figures 5A,B). In the HTK-Flush   and HTK + Ruxo groups, nitrotyrosine levels were comparable to those in the Control group. The expression of eNOS showed only minimal differences, with slightly lower expression in the HTK group (Figures 5A,B).

**Figure 5:**
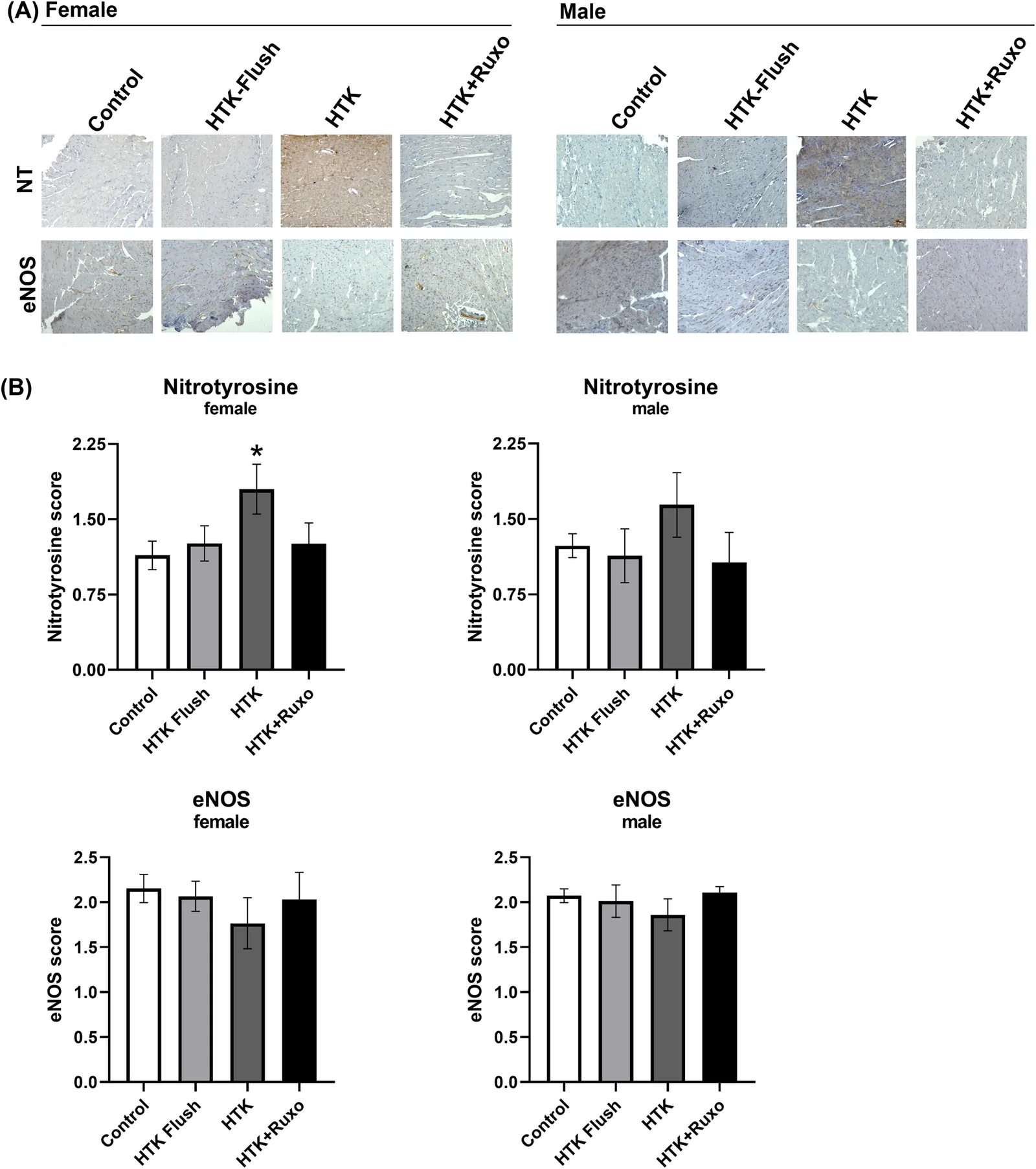
Immunohistochemical staining. **(A)** Representative pictures of NT and eNOS. **(B)** Comparison of NT and eNOS expression among groups. eNOS: Endothelial nitric oxide synthase. HTK: Histidine-Tryptophane-Ketoglutarate. NT: Nitrotyrosine. Ruxo: Ruxolitinib. *N* = 5/group and sex. *p < 0.05.

## Discussion

The functional and molecular analyses demonstrated that a single flush of donor hearts with HTK solution, without subsequent storage, does not affect ventricular function, the JAK2/STAT3 pathway, nitrosative stress, or eNOS expression. Consequently, the functional decline in hearts from both sexes after SCS mainly refers to cold storage. The expected and profound functional decline reflects that hearts donated from older donors are less resilient to SCS and is in line with the higher rate of PGD after transplantation of hearts of older donors (2). The potential impact of sex on the occurrence of ventricular dysfunction in hearts donated from old donors could not be demonstrated in the present work, and is also likely to be part of a complex interplay of donor/recipient sex match/mismatch. Fortunately, ruxolitinib during SCS with HTK resulted in complete maintenance of ventricular contractility and relaxation in hearts from female old donors, and partial but improved maintenance in hearts from male old donors. Considering that the interaction of treatment and sex was not significant, a proven effect of sex on the effect of ruxolitinib was not apparent in the present study. Future studies beyond the explorative level with a larger group size could be helpful to address possible sex-dependent interactions.

Considering that we hypothesized that SASP-related cytokines are an important contributor to the functional decline of hearts in this context, relevant cytokines, such as IL-1*α*, IL-10, TNF-α, and IL-7, were released in greater amounts in the HTK group than in the HTK + Ruxo group in the hearts of female mice. However, these cytokines were released at lower or undetectable concentrations in hearts from male mice in both the HTK and HTK + Ruxo groups. In hearts from male mice, the phosphorylation of JAK2 was not induced. Consequently, it is reasonable that pJAK2 was also not reduced in the HTK + Ruxo group in female hearts. However, in hearts from male mice, JAK2 phosphorylation was induced; consequently, in the ruxolotinib-treated hearts of male donors, pJAK2 was lower than in the HTK group. Surprisingly, contractility was not positively affected by the altered JAK2 regulation, which also did not result in changes in STAT3 regulation. It should also be taken into account that pSTAT3 levels were comparable between HTK and HTK + Ruxo in female mouse hearts, which does not account for the differences in cytokine concentrations.

Additionally, at higher concentrations, ruxolitinib can inhibit tyrosine kinase 2 (TYK2). However, the local concentration of ruxolitinib, independent of the perfusate concentration applied, is unknown and likely lower. The differing cytokine concentrations between HTK and HTK + Ruxo in female hearts suggest that SASP-associated cytokines are specifically involved in the functional differences between the groups. Nevertheless, other mechanisms beyond a modification of JAK/STAT regulation were likely involved. It should also be taken into consideration that phosphorylation can be transient and might not be visible after a longer time, especially when small amounts of phosphorylated or non-phosphorylated proteins are present.

We also investigated Akt levels, a member of pathways important for cellular survival, metabolism, and homeostasis, which were higher in the HTK + Ruxo group than in the HTK group in hearts from male donors. However, we did not find substantial differences among groups in hearts from female mice. Thus, the Akt pathway did not appear to be involved in the functional group differences observed in hearts from female mice. In hearts from male mice, the involvement of Akt also requires further verification, given that pAKT levels were elevated in the HTK-flush group.

Our results also showed that functional deterioration in the HTK group was accompanied by increased nitrosative stress, and that adding ruxolitinib to HTK reduced nitrosative stress in hearts from old donors of both sexes, potentially improving myocardial protection. No scavenging properties for reactive nitrogen species are known from ruxolitinib. However, IL-1*α* and TNF-α, which were decreased in the HTK + Ruxo group, are involved in the production of reactive nitrogen and oxygen species (10, 11). Consequently, the reduced nitrosative stress could have resulted from reduced release of the respective cytokines. Considering that a well-preserved coronary endothelium is also important for graft function, we measured eNOS, which, however, appears to play a minor role in this context, as its expression was comparable across all groups.

While the beneficial effect of ruxolitinib during SCS is apparent, the underlying mechanisms are complex and warrant further investigation. SASP-related cytokines appear to be involved, at least in the hearts of older female donors. However, their role in hearts from old male mice warrants further exploration. In future work, it should also be taken into consideration that not all older individuals are in the same health condition, nor do they present the same level of fitness, resilience, or frailty, nor do their organs. Potentially, ruxolitinib addresses an additional mechanism beyond its classic mode of action (JAK inhibition), thereby improving donor heart preservation, especially in hearts procured from female donors.

Higher levels of relevant SASP-related cytokines in females can theoretically be attributed to the male-female health-survival paradox. Accordingly, it was shown that females tend to live longer but in worse health and with greater frailty than men, even when the same age is compared between the two sexes. Women also tend to show a greater decline in intrinsic capacity and resilience with age (14–16). Lower resilience and higher frailty are associated with higher levels of SASP-related proinflammatory cytokines, which is consistent with the elevated levels of IL-1*α*, IL-10, TNF-α, and IL-7 in the first coronary effluent after SCS in the present study. Consequently, future studies should also focus on sex-dependent effects of frailty to address the mentioned assumptions.

### Limitations

This study has limitations. First, the results are based on animal experiments rather than human data. However, this is based on the fact that new methods enter clinical studies only after successful basic science studies. Second, we did not perform an orthotopic HTX, which limited the interpretability of our *ex vivo* reperfusion and functional analysis. *In vivo* reperfusion, such as after orthotopic HTX, more closely reflects the clinical procedure.

Nevertheless, the recipient's condition and age also significantly affect graft survival and may introduce bias into the results (12, 13). Thus, our experimental protocol allows the isolated investigation of the donor-related aspects. Third, considering the relatively small number of animals used in this study, we suggest interpreting the results as exploratory. Fourth, we did not perform randomization. However, laboratory mice are very comparable when bred in the same research facility. Future experiments should also investigate whether the observed efficacy of ruxolitinib can be transferred to hearts donated from young animals.

## Conclusion

Based on our data, the senomorphic agent ruxolitinib leads to a major functional improvement in cardiac allografts procured from old donors after SCS when added to the preservation solution, which in this case was HTK. This effect might be more pronounced in hearts donated by female donors. The observed effect could lead to reduced rates of PGD after orthotopic transplantation of hearts donated by older donors. If the results from the present study could be translated into a model of orthotopic HTX, another milestone towards clinical transplantation of hearts from older donors could be made. Thus, the organ shortage could also be partially compensated. Nevertheless, much work is still needed to unravel underlying mechanisms, considering the complex interplay of aging, SASP, and IRI in the heart with regard to sex-dependent differences.

## Data Availability

The raw data supporting the conclusions of this article will be made available by the authors, without undue reservation.

## References

[1] Khush KK Potena L Cherikh WS Chambers DC Harhay MO Hayes D. The international thoracic organ transplant registry of the international society for heart and lung transplantation: 37th adult heart transplantation report-2020; focus on deceased donor characteristics. J Heart Lung Transplant. (2020) 39(10):1003–15. 10.1016/j.healun.2020.07.010 32811772 PMC7737223

[2] Jernryd V Stehlik J Metzsch C Lund LH Gustav Smith J Andersson B. Donor age and ischemic time in heart transplantation—implications for organ preservation. J Heart Lung Transplant. (2025) 44(3):364–75. 10.1016/j.healun.2024.10.030 39491603

[3] Buchan TA Moayedi Y Truby LK Guyatt G Posada JD Ross HJ. Incidence and impact of primary graft dysfunction in adult heart transplant recipients: a systematic review and meta-analysis. J Heart Lung Transplant. (2021) 40(7):642–51. 10.1016/j.healun.2021.03.015 33947602

[4] Schroder JN Patel CB DeVore AD Casalinova S Koomalsingh KJ Shah AS. Increasing utilization of extended criteria donor hearts for transplantation: the OCS heart EXPAND trial. JACC Heart Fail. (2024) 12(3):438–47. 10.1016/j.jchf.2023.11.015 38276933

[5] Singh SSA Dalzell JR Berry C Al-Attar N. Primary graft dysfunction after heart transplantation: a thorn amongst the roses. Heart Fail Rev. (2019) 24(5):805–20. 10.1007/s10741-019-09794-1 31020451 PMC6697758

[6] Nian M Lee P Khaper N Liu P. Inflammatory cytokines and postmyocardial infarction remodeling. Circ Res. (2004) 94(12):1543–53. 10.1161/01.RES.0000130526.20854.fa 15217919

[7] Shawky AM Almalki FA Abdalla AN Abdelazeem AH Gouda AM. A comprehensive overview of globally approved JAK inhibitors. Pharmaceutics. (2022) 14(5):1001. 10.3390/pharmaceutics14051001 35631587 PMC9146299

[8] Xu M Tchkonia T Ding H Ogrodnik M Lubbers ER Pirtskhalava T. JAK inhibition alleviates the cellular senescence-associated secretory phenotype and frailty in old age. Proc Natl Acad Sci U S A. (2015) 112(46):E6301–10. 10.1073/pnas.1515386112 26578790 PMC4655580

[9] Saemann L Naujoks P Hartrumpf L Pohl S Simm A Szabó G. Sex-specific protection of endothelial function after vascular ischemia/reperfusion injury by the senomorphic agent ruxolitinib. Int J Mol Sci. (2023) 24(14):11727. 10.3390/ijms241411727 37511486 PMC10381013

[10] Ding AH Nathan CF Stuehr DJ. Release of reactive nitrogen intermediates and reactive oxygen intermediates from mouse peritoneal macrophages. Comparison of activating cytokines and evidence for independent production. J Immunol. (1988) 141(7):2407–12. 10.4049/jimmunol.141.7.2407 3139757

[11] Meier B Radeke HH Selle S Younes M Sies H Resch K. Human fibroblasts release reactive oxygen species in response to interleukin-1 or tumour necrosis factor-alpha. Biochem J. (1989) 263(2):539–45. 10.1042/bj2630539 2556998 PMC1133461

[12] Iyanna N Hong Y Hess NR Ziegler LA Dorken-Gallastegi A Hickey GW. Donor and recipient age influence outcomes following orthotopic heart transplantation in the 2018 US heart allocation system. Transplantation. (2025) 109(3):549–57. 10.1097/TP.0000000000005194 39192472

[13] Hsich E Singh TP Cherikh WS Lewis A Perch M Hayes D. International experience of donation after circulatory death for heart transplantation: a special report from the international society for heart and lung transplantation thoracic organ transplant registry. J Heart Lung Transplant. (2025) 44(9):1365–74. 10.1016/j.healun.2025.04.009 40300678

[14] Quintás-Cardama A Vaddi K Liu P Manshouri T Li J Scherle PA. Preclinical characterization of the selective JAK1/2 inhibitor INCB018424: therapeutic implications for the treatment of myeloproliferative neoplasms. Blood. (2010) 115(15):3109–17. 10.1182/blood-2009-04-214957 PMC3953826 20130243

[15] Gordon EH Peel NM Samanta M Theou O Howlett SE Hubbard RE. Sex differences in frailty: a systematic review and meta-analysis. Exp Gerontol. (2017) 89:30–40. 10.1016/j.exger.2016.12.021 28043934

[16] Li Y Zhang T Li M Shen R Wang X Zhuo C. Trajectories of intrinsic capacity decline and related factors in old persons: a 15-year community-based cohort study in Beijing. J Nutr Health Aging. (2025) 29(5):100526. 10.1016/j.jnha.2025.100526 40048878 PMC12180025

