# Supplementary material for: Improved preservation of hearts from old donors during static cold storage by the senomorphic agent ruxolitinib
Source: Front Transplant. 2026 Oct 28;5:1917058. doi: 10.3389/frtra.2026.1917058 (PMC13752187; doi:10.3389/frtra.2026.1917058)
Supplement: Supplementary file 1 [file Table1.docx]

**Supplemental**

*SASP-related cytokine expression*

We collect the very first coronary affluent after the onset of reperfusion. A standardized volume of 500 µl was snap-frozen in liquid nitrogen and stored at -80 °C for further analysis. The cytokines were measured as previously described ^10^. We performed a customized multiplex magnetic bead assay (ProcartaPlex Mouse and Rat Mix&Match Panels, Thermo Fisher Scientific, Waltham, USA) with the incubation supernatant to quantify the myocardial SASP-related expression. We incubated 50 µl of the supernatant over night with the magnetic beads added to the plate. The remaining steps were conducted according to the manufacturer's instructions, consisting of washing steps, the addition of a detection antibody, streptavidin-RPE, and a reading buffer. All incubations were performed with agitation. We included all out-of-range low values as a concentration of 0 ng/ml in the analysis.

*Tissue collection*

After the second functional evaluation, the hearts were flushed with an ice-cold saline solution. Samples of left-ventricular myocardial tissue were frozen in liquid nitrogen and stored at -80°C until further analysis. In addition, we conserved tissue samples in paraformaldehyde solution and embedded them in paraffin to cut into 1.5 µm thick slices, which were placed on slides.

*Western Blotting*

Frozen myocardial tissue samples were lysed in RIPA buffer (#9806, Cell Signaling Technology, Leiden The Netherlands) and phosphatase inhibitors cocktail (11836153001, Roche, Pensberg, Germany). Tissue homogenization was performed for 30 min, followed by centrifugation at 13.000 rpm for 20 min at 4°C. The protein concentration was measured using a BCA assay (Pierce, 232265, USA). Then the proteins were deactivated at 95°C for 5 min. 50 µg protein per slot were mixed with  equivalent volumes of SDS buffer (Tris, glycine, SDS) and  subjected to electrophoresis using 6–10% SDS–polyacrylamide gels. Electrophoresis (Mini-PROTEAN Tetra, BioRad, Feldkirchen, Germany) was performed at 80 V for 30 minutes followed by 110 V until done, using a running buffer containing Tris, glycine and SDS. Then PCVD membrane was reactivated with methanol for 30 sec. Proteins were transferred to PVDF (IPVH00010, Immobilon-P, Millipore, Darmstadt, Germany) membranes using a wet transfer system (Mini Trans-Blot® Cell, Biorad, Feldkirchen, Germany) at 350 mA for 1 hours in ice-cold transfer buffer (Tris, Glycine, methanol). Then, the membrane was placed in TBST buffer containing Tris, NaCl and Tween. Blocking was performed for 1 h with 5% BSA or milk depending on the antibody. Membranes were then incubated with primary antibodies overnight at 4°C with agitation. After three additional 5-minute washes with TBST, the membranes were incubated with the corresponding secondary antibodies for 2 h. Signal detection was performed with a WesternBright Quantum system (Advansta, San Jose, USA). Signal intensities were normalized to vinculin within each subgroup. Detailed information on western blotting and antibodies (Table S1) are shown in the Supplemental.

**Supplementary Table S1**: Primary and secondary antibodies for Western blotting.

| **Prim. AB** | **Order. No./Company** | **Dilution** | **Clone** | **sek. AB** | **Size** |
| --- | --- | --- | --- | --- | --- |
| Anti-mTOR | 66888-1-Ig/  Proteintech, Planegg-Martinsried, Germany | 1: 5000 | Monocl. | Anti mouse | 250kDa |
| Anti-p-mTOR | #5536/  CellSignaling, Leiden The Netherlands | 1:1000 | Monocl. | Anti rabbit | 289kDa |
| Anti-Vinculin | 66305-1-Ig/  Proteintech, Planegg-Martinsried, Germany | 1:50000 | Monocl. | Anti mouse | 117kDa |
|  |  |  |  |  |  |
| Anti-STAT3 | #30835/ CellSignaling, Leiden The Netherlands | 1:1000 | Monocl. | Anti rabbit | 79,86kDa |
| Anti-p-STAT3 | #9131/ CellSignaling, Leiden The Netherlands | 1:1000 | Polycl. | Anti rabbit | 86kDa |
| Anti-GAPDH | 60004-1-Ig/ Proteintech, Planegg-Martinsried, Germany | 1:50000 | Monocl. | Anti mouse | 37kDa |
|  | | | | | |
| AKT(pan) | #2920/  CellSignaling, Leiden The Netherlands | 1:2000 | Monocl. | Anti mouse | 60kDa |
| p-AKT | #4058/  CellSignaling, Leiden The Netherlands | 1:1000 | Monocl. | Anti rabbit | 60kDa |
| Anti-GAPDH | 60004-1-Ig/  Proteintech, Planegg-Martinsried, Germany | 1:50000 | Monocl. | Anti mouse | 37kDa |
|  | | | | | |
| JAK 2 | #3230  CellSignaling, Leiden The Netherlands | 1:1000 | Monocl. | AR | 125kDa |
| p-JAK 2 | #3776  CellSingnaling, Leiden The Netherlands | 1:1000 | Monocl. | AR | 125kDa |
|  | | | | | |
| Anti mouse HRP | #7076  CellSignaling, Leiden The Netherlands | 1:1000 |  |  |  |
| Anti Rabbit  HRP | #7074  CellSignaling, Leiden The Netherlands | 1: 1000 |  |  |  |

*Immunohistochemical staining*

Immunohistochemical staining of nitrotyrosine (HM5001, Hycult Biotech, Uden, The Netherlands, 1:200 dilution, Citrat pH6.0) a marker of nitrosative stress, endothelial nitric oxide synthase (eNOS, AB30007, Abcam, Cambridge, UK, 1:100 dilution, Tris-EDTA pH9.0).
